# Visual Acuity by Decade in 139 Males with *RPGR*-Associated Retinitis Pigmentosa

**DOI:** 10.1016/j.xops.2023.100375

**Published:** 2023-07-24

**Authors:** Samantha R. De Silva, Hwei Wuen Chan, Aditi Agarwal, Andrew R. Webster, Michel Michaelides, Omar A. Mahroo

**Affiliations:** 1 UCL Institute of Ophthalmology, University College London, London, United Kingdom; 2 Genetics Service, Moorfields Eye Hospital, London, United Kingdom; 3 Section of Ophthalmology, King's College London, St Thomas' Hospital Campus, London, United Kingdom; 4 Physiology, Development and Neuroscience, University of Cambridge, Cambridge, United Kingdom

**Keywords:** Inherited retinal degeneration, Retinitis pigmentosa, RPGR, Visual acuity

Inherited retinal diseases are a leading cause of blindness in working age adults in many countries. X-linked retinitis pigmentosa (RP) accounts for a significant proportion, with the majority associated with pathogenic variants in the *RPGR* gene.^1^ Although currently untreatable, 2 phase III gene therapy trials are ongoing (NCT04850118 and NCT04671433). Patients experience debilitating restriction of the visual field,^2^ but a key additional milestone is the subsequent loss of central vision. We collected visual acuity (VA) data from the large patient cohort with molecularly proven *RPGR*-associated RP in our center, aiming to quantify average VA for each decade, specifically identifying the decade in which this declined to 1.0 logarithm of the minimum angle of resolution (logMAR) or worse. We also investigated correlation between right and left eye acuities for all visits.

The analysis was restricted to male patients and to those with the more common rod-cone dystrophy/RP phenotype. The diagnosis of rod-cone dystrophy was made on the basis of visual symptoms (typically night vision difficulties followed by peripheral visual field loss), features on clinical examination and retinal imaging (including bone spicule pigmentation or predominantly peripheral retinal atrophy), and, if available, electrodiagnostic testing. Those with the later-presenting macular dystrophy, cone dystrophy, or cone-rod dystrophy phenotypes were not included in the main study. Best-recorded VA in each eye was converted to logMAR units (with qualitative acuities treated using previously published conversions, described in the legend to Fig 1).^3^^,^^4^ For quantification of average acuity per decade, if patients had multiple visits within the same decade, only 1 of these visits (the first) was included, so that no patient was included more than once in each decade. The study had relevant institution review board approval (Moorfields Eye Hospital and the Northwest London Research Ethics Committee); the study adhered to the Declaration of Helsinki and patients gave informed consent.

**Figure 1:**
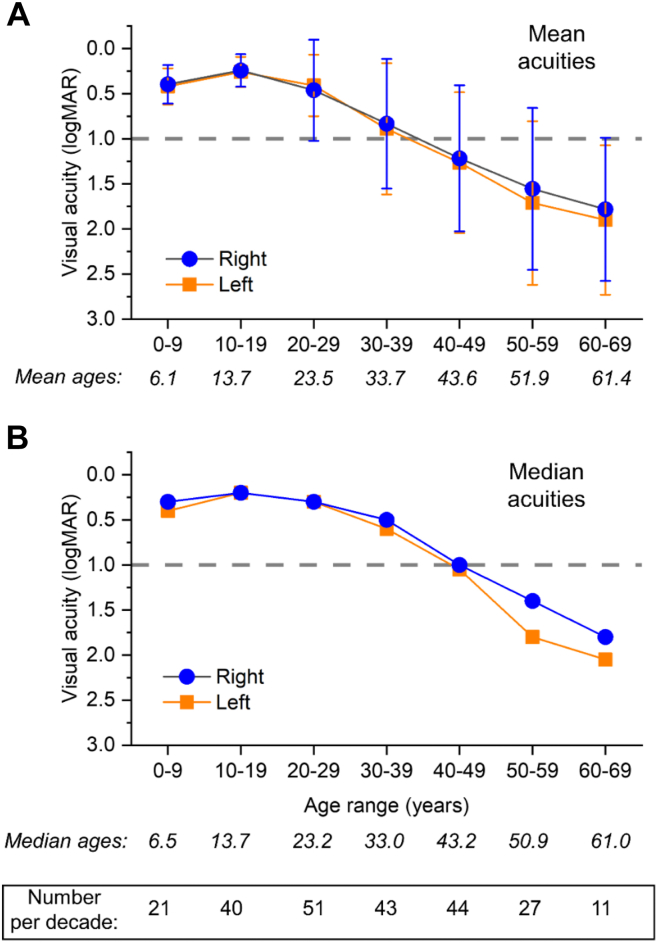
Average acuities per age decade in 139 male patients with molecularly proven *RPGR*-associated retinitis pigmentosa. Visual acuities were converted from the original measurement method to logarithm of the minimum angle of resolution (logMAR) units (with qualitative acuities of counting fingers, hand movements, light perception, and no light perception, defined as 1.9, 2.3, 2.7, and 3.0, respectively). For patients in whom visual acuities were recorded more than once in a particular decade, data from only 1 visit (the first) were included in that decade, so that no patient was included more than once in each decade. **A,** points plot mean acuities (error bars denote standard deviations) for right and left eyes. Numbers in italics below the x-axis give the mean age of patients included in the particular decade. **B,** points plot median acuities for right and left eyes. Numbers in italics below the x-axis give median ages of patients included in the particular decade. The box at the bottom of the figure gives numbers of patients included in each decade.

Data were analyzed from 771 patient visits (139 patients). Across all visits, mean (standard deviation [SD]) patient age was 32 (15) years and the median age was 33 years. Mean (SD) logMAR VA was 0.82 (0.81) and 0.86 (0.82) for right and left eyes, respectively; medians were 0.5 and 0.5, respectively. Figure 1 shows average (mean and median) VAs for right and left eyes by decade. Numeric values are given in Table S1 (available at www.ophthalmologyscience.org). Distributions of VAs in each decade are shown in the plots in Figure S2 (available at www.ophthalmologyscience.org). As well as shifting toward worse acuities in the older decades, the distributions become more dispersed, and some eyes (a minority) had VAs worse than 1.0 logMAR in the third and fourth decades. Correlations of acuity with age were 0.66 and 0.67 for right and left eyes, respectively (*P* < 0.001).

Figure S3 (available at www.ophthalmologyscience.org) shows right and left eye acuities across all visits. The coefficient of interocular correlation was 0.88 (*P* < 0.001). The high interocular correlation was expected, consistent with largely symmetric disease in men.^5^ However, significant interocular differences were also seen, particularly for worse VAs: the mean (SD) absolute interocular difference was 0.12 (0.22) for patients with acuities better than 1.0 logMAR (averaged across both eyes); the mean (SD) difference was 0.43 (0.54) for those with 1.0 logMAR or worse. This is relevant when fellow eyes serve as controls in therapy trials.

When quantifying VA by decade in our cohort of > 130 patients (larger than previously published cohorts), we found that both mean and median VA reached 1.0 logMAR or worse in the fifth decade for both right and left eyes, and for the better-seeing eye; thus, the majority of patients had vision of this level or worse from this decade onward. The mean age of patients whose VA was 1.0 logMAR or worse in their better-seeing eye was 44 years (median, 45 years). Our results are largely consistent with data from previous *RPGR*-associated rod-cone dystrophy cohorts, with 1 study reporting the median age to legal blindness at the age of 45 years (n = 113)^6^ and another reporting a 20% probability of reaching the threshold of legal blindness at the age of 40 years (n = 52).^2^

There were 16 additional patients in our cohort with *RPGR*-associated cone or cone-rod dystrophy (excluded from the above analysis). Data were available from 102 patient visits: across all visits, mean (SD) patient age was 45 (10) years; median age was 47.5 years. Mean (SD) logMAR VA was 1.1 (0.18) and 1.1 (0.44) for right and left eyes, respectively; medians were 1.0 and 1.0, respectively. These findings are consistent with patients with this phenotype presenting at an older age than those with rod-cone dystrophy (the median age of patients with rod-cone dystrophy was 33 years). In contrast to the rod-cone phenotype, patients with the cone or cone-rod dystrophy usually present with VA reduction. Given the difference in presentation and disease natural history, data from these patients were not included in our main analysis. Also, patients with this phenotype are not currently being recruited to the *RPGR* gene therapy trials.

Limitations of this study include its retrospective nature and that visual field data were not available. Most patients with the RP phenotype have extensive visual field loss, resulting in severe sight impairment, before the fifth decade. However, the timing of loss of central vision is an important additional feature of the natural history which has a marked stepwise impact on functional vision, and our finding that there is greater interocular asymmetry at worse levels of acuity is important for informing clinical trial design. These findings will inform counseling of patients and evaluation of net benefit of new treatments, including helping define the potential window for therapeutic intervention.

## Supplementary Data

Supplemental Figure 1 Distributions of acuities per age decade in 139 male patients with molecularly proven *RPGR*-associated retinitis pigmentosa. Visual acuities were converted from the original measurement method to LogMAR units. For patients in whom visual acuities were recorded more than once in a particular decade, data from only one visit (the first) were included in that decade, so that no patient was included more than once in each decade. *A*, Boxplots of acuities in each decade for right eyes. *B*, Boxplots of acuities in each decade for left eyes. *C*,*D*, Violin plots of acuities per decade for right and left eyes respectively.

Supplemental Figure 2 Right and left visual acuities in male patients with molecularly proven *RPGR*-associated retinitis pigmentosa across all visits. Visual acuities were converted from the original measurement method to LogMAR units (with qualitative acuities of counting fingers, hand movements, light perception, and no light perception, defined as 1.9, 2.3, 2.7, and 3.0, respectively). The grey diagonal line represents equal acuities in both eyes. Tight agreement is seen between eyes for patients with better acuities; greater differences are seen in those with worse acuity. Overall coefficient of interocular correlation (Pearson and Spearman) was 0.88 (*p*<0.0001).

Supplemental Table 1 Mean (SD) and median visual acuities per decade.
