# Supplementary material for: Visual Acuity by Decade in 139 Males with RPGR-Associated Retinitis Pigmentosa
Source: Ophthalmol Sci. 2023 Jul 24;4(2):100375. doi: 10.1016/j.xops.2023.100375 (PMC10587616; doi:10.1016/j.xops.2023.100375)
Supplement: Supplemental Figure 1 — Distributions of acuities per age decade in 139 male patients with molecularly proven RPGR-associated retinitis pigmentosa. Visual acuities were converted from the original measurement method to LogMAR units. For patients in whom visual acuities were recorded more than once in a particular decade, data from only one visit (the first) were included in that decade, so that no patient was included more than once in each decade. A, Boxplots of acuities in each decade for right eyes. B, Boxplots of acuities in each decade for left eyes. C,D, Violin plots of acuities per decade for right and left eyes respectively. [file mmc1.pdf]

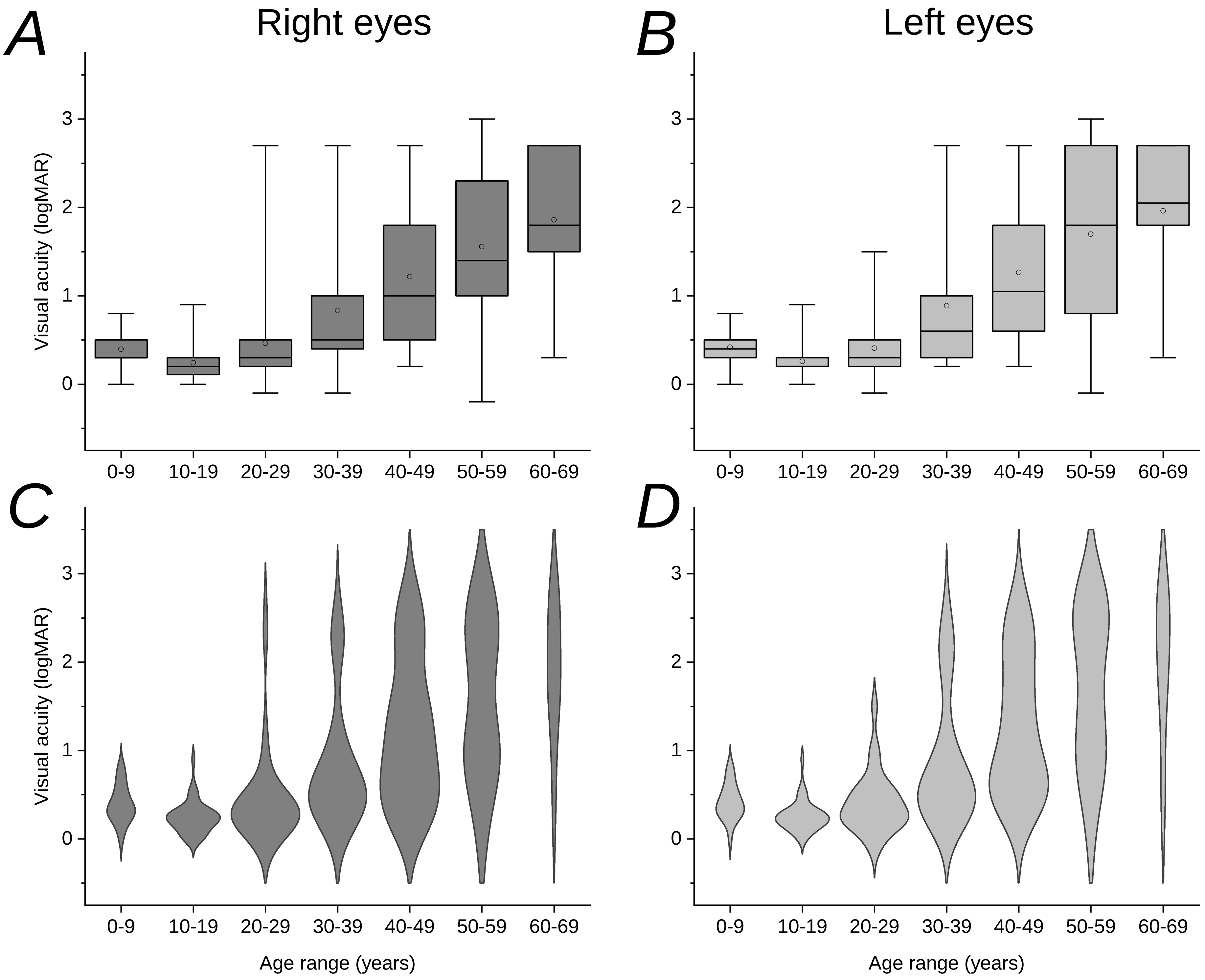

Supplemental Figure 1. Distributions of acuities per age decade in 139 male patients with molecularly proven *RPGR*-associated retinitis pigmentosa. Visual acuities were converted from the original measurement method to LogMAR units. For patients in whom visual acuities were recorded more than once in a particular decade, data from only one visit (the first) were included in that decade, so that no patient was included more than once in each decade. *A*, Boxplots of acuities in each decade for right eyes. *B*, Boxplots of acuities in each decade for left eyes. *C,D*, Violin plots of acuities per decade for right and left eyes respectively.
