# Supplementary material for: Visual Acuity by Decade in 139 Males with RPGR-Associated Retinitis Pigmentosa
Source: Ophthalmol Sci. 2023 Jul 24;4(2):100375. doi: 10.1016/j.xops.2023.100375 (PMC10587616; doi:10.1016/j.xops.2023.100375)
Supplement: Supplemental Figure 2 — Right and left visual acuities in male patients with molecularly proven RPGR-associated retinitis pigmentosa across all visits. Visual acuities were converted from the original measurement method to LogMAR units (with qualitative acuities of counting fingers, hand movements, light perception, and no light perception, defined as 1.9, 2.3, 2.7, and 3.0, respectively). The grey diagonal line represents equal acuities in both eyes. Tight agreement is seen between eyes for patients with better acuities; greater differences are seen in those with worse acuity. Overall coefficient of interocular correlation (Pearson and Spearman) was 0.88 (p<0.0001). [file mmc2.pdf]

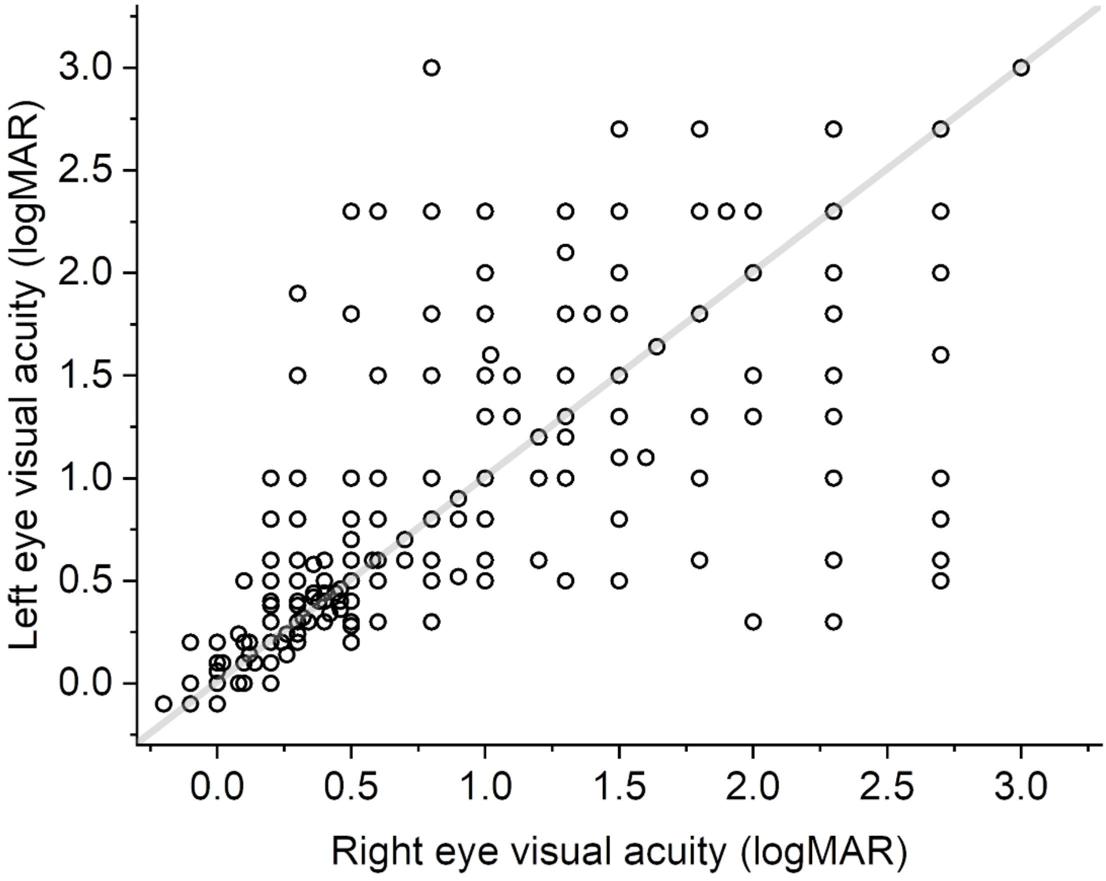

Supplemental Figure 2. Right and left visual acuities in male patients with molecularly proven *RPGR*-associated retinitis pigmentosa across all visits. Visual acuities were converted from the original measurement method to LogMAR units (with qualitative acuities of counting fingers, hand movements, light perception, and no light perception, defined as 1.9, 2.3, 2.7, and 3.0, respectively). The grey diagonal line represents equal acuities in both eyes. Tight agreement is seen between eyes for patients with better acuities; greater differences are seen in those with worse acuity. Overall coefficient of interocular correlation (Pearson and Spearman) was 0.88 (p<0.0001).
