# Supplementary material for: Visual Acuity by Decade in 139 Males with RPGR-Associated Retinitis Pigmentosa
Source: Ophthalmol Sci. 2023 Jul 24;4(2):100375. doi: 10.1016/j.xops.2023.100375 (PMC10587616; doi:10.1016/j.xops.2023.100375)
Supplement: Supplemental Table 1 — Mean (SD) and median visual acuities per decade. [file mmc3.pdf]

Supplemental Table 1. Mean (SD) and median visual acuities per decade.

Age decade (y)	Number of patients	Mean age (y)	Median age (y)	Mean (SD) acuity (logMAR)		Median acuity (logMAR)	
				Right	Left	Right	Left
0-9	21	6.1	6.5	0.40 (0.21)	0.42 (0.20)	0.30	0.40
10-19	40	13.7	13.7	0.24 (0.18)	0.26 (0.17)	0.20	0.20
20-29	51	23.5	23.3	0.46 (0.56)	0.41 (0.34)	0.30	0.30
30-39	43	33.7	33.0	0.83 (0.72)	0.89 (0.73)	0.50	0.60
40-49	44	43.6	43.2	1.22 (0.81)	1.26 (0.78)	1.00	1.05
50-59	27	51.9	50.9	1.56 (0.90)	1.71 (0.91)	1.40	1.80
60-69	11	61.4	61.0	1.78 (0.79)	1.90 (0.83)	1.80	2.05
